# Nontuberculous mycobacterial infection after frontalis sling surgery using silicone rod

**DOI:** 10.1007/s12348-012-0073-y

**Published:** 2012-04-05

**Authors:** Batriti Walang, Suryasnata Rath, Savitri Sharma

**Affiliations:** 1 Ophthalmic Plastic & Reconstructive Surgery, Orbit, and Ocular Oncology Service, L V Prasad Eye Institute, Bhubaneswar, Orissa India 751024; 2 Microbiology Service, L V Prasad Eye Institute, Bhubaneswar, India 751024

**Keywords:** Nontuberculous mycobacteria, Frontalis sling surgery, Silicone

## Abstract

**Purpose:**

The purpose of this study is to report a case of nontuberculous mycobacterial infection after frontalis sling surgery.

**Method:**

A 65-year-old man presented with bilateral painful, erythematous lesions in the brow and upper eyelids. He had a history of frontalis sling surgery for myopathic ptosis 2 years back and all lesions were found localized to the tract of the silicone rod used in the previous frontalis sling surgery.

**Result:**

Incision and drainage of the lesions with microbiological analysis revealed significant growth of coagulase negative staphylococcus and *Mycobacterium fortuitum*. Sensitivity-based antibiotic treatment with intravenous amikacin was started, but poor response necessitated eventual explantation of both silicone rods for relief of symptoms. Culture of the explanted rods revealed similar results of *M. fortuitum* infection. Five months after the acute presentation, the patient is asymptomatic.

**Conclusion:**

Nontuberculous mycobacterial infection may be a delayed onset complication in frontalis sling surgery using silicone rods.

## Introduction

Frontalis sling surgery is the treatment option for myopathic blepharoptosis with poor levator muscle action [1]. Infections associated with the silicone rod after frontalis sling surgery have been reported [2]. Nontuberculous mycobacterial infections in the periocular region are rare and are usually caused by organisms belonging to Runyon group IV including *Mycobacterium chelonae* and *Mycobacterium fortuitum* [3]. Chang et al. reported six cases of nontuberculous mycobacterial infection and found an association with nasolacrimal duct obstruction, implantation of foreign body, history of recent surgery, and immunosuppression [3]. Mauriello found implantation of a foreign body in 5 out 13 patients of nontuberculous mycobacterial infections of the periocular region [4]. Treatment of nontuberculous mycobacterial infections can be difficult because of their multi-drug resistance [3]. Treatment usually involves surgical debridement with removal of infected foreign body and a prolonged course of antibiotics like amikacin, clarithromycin, ciprofloxacin, and doxycycline [3]. We report a rare case of nontuberculous mycobacterial infection of the silicone rod after frontalis sling surgery.

## Case report

**Fig. 1:**
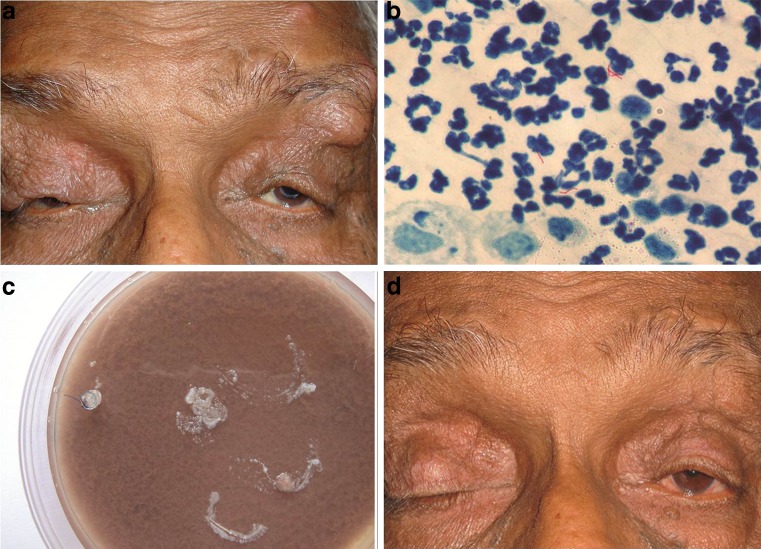
**a** An apparently healthy 62-year-old man presented with painful, erythymatous nodules of bilateral brow and upper lids two years after frontalis sling surgery for severe myopathic ptosis. Incision and drainage with microbiological examination revealed infection by *Staphylococcus* species and *Mycobacterium fortuitum*. **b** Microscopic picture of the purulent material showing long, slender acid fast bacilli along with polymorphonuclear cells (Ziehl Neelsen stain, total magnification ×1,000). **c** Sheep blood chocolate agar inoculated with purulent material along with silicone tube (left eye brow after 11 days of treatment) shows confluent growth of cream colored, opaque, medium size colonies of *Staphylococcus aureus* and tiny, semi-opaque colonies of *Mycobacterium fortuitum* (incubation: 4 days, 5 % CO_2_, 37 °C). **d** Five months after sensitivity-based antibiotic treatment and explantation of bilateral silicone rods, the patient showed resolution of symptoms. The left upper eyelid maintained an elevated position after silicone rod explantation

A 65-year-old apparently healthy male presented with complaints of pain and swelling of both eyelids of 2 months duration. He had undergone cataract and frontalis sling surgery for myopathic blepharoptosis in both eyes 2 years prior to presentation. The best corrected visual acuity for distance was 20/400 in right eye and 20/25 in the left eye. Examination of face showed multiple, erythematous nodules involving both upper eyelids and the brow. All nodules were localized to the scars from the previous ptosis surgery with a few showing signs of suppuration (Fig. 1a). The nodules in the upper eyelids were preseptal and mobile. Ocular motility was limited in all directions and diplopia test was negative. Anterior segment examination was unremarkable except for the pseudophakia in both eyes. The posterior segment showed hypertensive retinopathy in both eyes and a scar at the macula in the right eye. Lacrimal passages were patent. Systemic workup was unremarkable and included a complete blood picture, chest X-ray and serological tests for HIV. The patient was clinically diagnosed to have chronic progressive external ophthalmoplegia status post bilateral frontalis sling and cataract surgery with multiple abscesses in forehead and upper eyelids. The patient underwent an incision and drainage of the abscesses in the brow and the purulent material was sent for microbiological examination. The smear showed the presence of acid fast bacilli on 20 % acid fast staining (Fig. 1b) and culture was significant for *Staphylococcus* sp. and *M. fortuitum* (Fig. 1c). KB disk method showed *Staphylococcus* sp. to be sensitive to all antibiotics. *M. fortuitum* showed sensitivity to amikacin and intermediate sensitivity to ciprofloxacin. The patient received intravenous amikacin (1 g/day, single dose) and ciprofloxacin ointment locally. Poor response to treatment necessitated exploration with explantation of the silicone rod sling in the right eye 10 days later. A repeat microbiological evaluation showed acid fast bacilli and significant growth of *M. fortuitum* on culture. As the isolate showed a similar antibiotic sensitivity pattern the patient was continued on intravenous amikacin (1 g/day). Regular kidney function tests were done to look for amikacin-related nephrotoxicity. There was definite improvement in symptoms and resolution of the abscesses. However, 11 days later, the patient suffered from another episode of pain and swelling in the left eye. The left eyebrow and eyelid were inflamed with an abscess in the forehead. A repeat exploration of the brow with drainage and silicone rod explantation was performed in the left eye. The patient showed a slow but definite improvement and received intravenous amikacin for 6 weeks. Five months after commencement of therapy the patient was asymptomatic and the left upper eyelid position was maintained (Fig. 1d).

## Discussion

Several authors have reported about nontuberculous mycobacterial infections after periocular surgery [3, 4]. The median time interval between prior surgery and onset of infection in these reports was 6 weeks (range 0.5–11 months) [3, 4]. In our patient, multiple erythematous nodules with suppuration along the tract of the silicone rod more than 2 years after surgery led to the suspicion and eventual diagnosis of nontuberculous mycobacteria. Some aspects in our patient need to be highlighted.

First is that a bilateral infection by nontuberculous mycobacteria involving the silicone rod after frontalis sling surgery has not been reported earlier. The lodgement of nontuberculous mycobacteria in the brow region in our case may be related to the persistent irritation from the silicone rod and its predeliction for fat [4, 5]. Sequestration of nontuberculous mycobacteria with fat allows its growth without detection by normal immunosurveillance [4, 5]. In our patient, sequestration of nontuberculous mycobacteria in the brow fat may have been responsible for delayed and bilateral infection involving the silicone slings. Further, Fitzgerald et al. found nontuberculous mycobacteria in 82 % of fat globules in their series of 71 cases [6].

Second is the chronic persistence of *M. fortuitum* infection after treatment with sensitive antibiotics. Complete resolution in our case occurred only after silicone slings were removed from both eyes.

Finally, removal of the silicone sling did not result in a recurrence of blepharoptosis. This phenomenon is postulated to be due to scarring and fibrosis along the tract of the silicone rod [7].

## Conclusion

Nontuberculous mycobacterial may be responsible for bilateral delayed infection after frontalis surgery with silicone slings in an immunocompetent adult. Surgical removal of infected slings and prolonged course of antibiotics are required for complete resolution.
